# Viewing health expenditures, payment and coping mechanisms with an equity lens in Nigeria

**DOI:** 10.1186/1472-6963-13-87

**Published:** 2013-03-09

**Authors:** Oforbuike Ewelukwa, Chima Onoka, Obinna Onwujekwe

**Affiliations:** 1 Health Policy Research Group, College of Medicine, University of Nigeria, Enugu, Nigeria; 2 Department of Community Medicine, College of Medicine, University of Nigeria, Enugu, Nigeria; 3 Department of Health Administration and Management, University of Nigeria, Enugu, Nigeria

**Keywords:** Equity, Socio-economic status, Payment mechanism, Health expenditure

## Abstract

**Background:**

This paper examines socio-economic and geographic differences in payment and payment coping mechanisms for health services in southeast Nigeria. It shows the extent to which the poor and rural dwellers disproportionally bear the burden of health care costs and offers policy recommendations for improvements.

**Methods:**

Questionnaires were used to collect data from 3071 randomly selected households in six communities in southeast Nigeria using a four week recall. The sample was divided into quintiles (Q1-Q5) using a socio-economic status (SES) index as well as into geographic groups (rural, peri-urban and urban). Tabulations and logistic regression were used to determine the relationships between payment and payment coping mechanisms and key independent variables. Q1/Q5 and rural/urban ratios were the measures of equity.

**Results:**

Most of the respondents used out-of-pocket spending (OOPS) and own money to pay for healthcare. There was statistically significant geographic differences in the use of own money to pay for health services indicating more use among rural dwellers. Logistic regression showed statistically significant geographic differences in the use of both OOPS and own money when controlling for the effects of potential cofounders.

**Conclusions:**

This study shows statistically significant geographic differences in the use of OOPS and own money to pay for health services. Though the SES differences were not statistically significant, they showed high equity ratios indicating more use among poor and rural dwellers. The high expenditure incurred on drugs alone highlights the need for expediting pro-poor interventions like exemptions and waivers aimed at improving access to health care for the vulnerable poor and rural dwellers.

## Background

Publicly financed health services have not been able to reach the poor in many developing countries [1], increasing the necessity of many people to use out-of-pocket spending (OOPS) to purchase health services. OOPS for health services has been shown to further impoverish the poor as well as exclude some of them from seeking health care [2]. While, recent studies show some gains in reduction of OOPS to less than 7% in a few low-income countries, it still takes up over 50% of the health financing of most of them mainly due to the lack of pre-payment mechanisms like Social Health Insurance [3,4]. The main consequences of absence of financial protection mechanisms have been reduction in access to quality health care, not seeking treatment, long-term poverty and indiscriminate use of drugs prescribed by quacks [5].

Despite the potential importance of user fees mostly paid as OOPS in developing countries in revenue generation, it has been shown to be the most regressive of all the financing mechanisms [6] leading to the clamour for its removal because of the huge barrier that it poses to accessing health care [7,8]. Studies have shown that the revenue generated through this means is too small to improve quality and that the structures are not in place to implement adequate exemption schemes to target the poorest households.

In 2010, private expenditure on health constituted 62% of overall health care spending in Nigeria. Of this figure, 95.3% came from OOPS [4]. Although user fee exemption currently features as an official policy measure of the federal government and of some state governments targeted at malaria cases, it is limited to under fives and pregnant women, and many poor and rural dwellers fall outside these groups. Also worth noting is that this policy has not been adopted by most states and it does not apply to private hospitals where more than 70% of malaria cases are treated. Furthermore, this policy does not differentiate between socio-economic status (SES) and geographic groups [9].

This study explores SES and geographic differences in the payment and payment coping mechanisms for health services in southeast Nigeria. It also analyses their impact on the access of poor and rural dwellers to health services. Payment mechanisms in this context refer to methods used by individuals to pay for health care and could be OOPS with reimbursement from employer, OOPS without reimbursement, health insurance, instalment or in-kind payments. Payment coping mechanisms refer to ways in which households respond to shocks from the payment mechanisms used to pay for health services e.g. use of own money, borrowed money, sale of assets, payment by subsidy/deferment/exemptions or by community support. For the purpose of this paper, OOPS is the same as OOPS without reimbursement from anyone, OOPS with reimbursement refers to those whose employers reimbursed their healthcare expenditure, use of own money as a coping mechanism refers to use of personal savings as opposed to subsidy, borrowing or contribution from family/friends to pay for healthcare.

## Methods

### Study area

This was a cross-sectional study conducted in two states (Anambra and Enugu) in southeast Nigeria. Anambra state has a population of 4,182,032; a land area of 4,416 km^2^ and its capital is at Awka while Enugu state has a population of 3,257,298, a land area of 7,618 km^2^ and has its capital at Enugu. The people of the two states are of the Igbo ethnic group, which is the third largest in the country and speak Igbo, English and a local variant of English called ‘pidgin’.

### Study sites

From each of the two states, a rural, a peri-urban and an urban area were randomly selected. The differences between the communities were based on the differences in population, levels of infrastructure and economic activities as shown by National Bureau of Statistics (NBC). Each of the selected communities has access to a health centre and was within a reasonable distance to a general hospital, as well as being served by some private hospitals, pharmacy shops, patent medicine dealers (PMD) and traditional healers.

### Nigerian health care system and financing

Health care delivery in Nigeria is provided by the government with a major input from the private sector which include private individuals, corporate bodies and churches that own and run organisations offering health care to the public. The government of Nigeria is divided into the federal, state and local governments with each of the three levels responsible for the funding and running of the three tiers of the health sector namely the tertiary, secondary and primary health centres respectively. The federal government through the Ministry of Health provides the overall policy guidelines and oversight functions for all arms of the health sector. The funding provided by each arm of the government is usually supplemented by money raised from OOPS from the public to make up for the short-fall [10].

A National Health Insurance Scheme (NHIS) was launched in Nigeria in 2005 to ensure adequate financial risk protection for the masses and to cushion the huge financial burden of health care cost borne by the government. NHIS is financed mainly from taxes, premiums and grants from the government as well as aid from non-governmental organisations and international and donor agencies [11]. Recent evidence shows that NHIS covers less than 5% of the population most of whom are federal civil servants, while other health insurance schemes like private health insurance and community-based health insurance (CBHI) cover less than 1% of the population [9].

### Data collection

A total of 3071 households were randomly selected from six communities using a sampling frame of households numbering system from the National Bureau of Statistics. An 80% power and 95% confidence interval were used to calculate adequate sample size to detect a 20% utilization of health services. In each of the households, informed consent was obtained and the head of the household was interviewed so that accurate information about the household characteristics and expenditure patterns could be obtained.

Weekly expenditure diaries were used to elicit information from respondents (who were mostly heads of households) on general household socio-demographics, ownership of assets like cars, bicycle, fridges, etc. as well as average weekly food expenditure, types of illnesses experienced in the past four weeks, where they first sought treatment, treatment cost and payment mechanisms for the health services they utilized. Quantitative ownership of assets (yes/no) was used as opposed to qualitative which may better differentiate the values of the assets. Ethical approval for the study was obtained from the Research Ethics Committee, University of Nigeria Teaching Hospital, Enugu, Nigeria.

### Data analysis

Tabulations and logistic regression were the data analytic methods. To determine the socio-economic differences, the data was used to construct an SES index using Principal Component Analysis (PCA) to categorise the population into quintiles based on asset ownership and per capita weekly food expenditure [12,13]. Variables used in computation of the SES index include radio, television, air-conditioner, bicycle, motorcycle, car, fridge, generator, electric fan as well as per capita average weekly food expenditure. Local knowledge and interview of some of the residents helped in choosing assets that were broad enough to show maximum variation in SE characteristics so as to avoid clumping and truncation [14]. PCA based on asset ownership and average weekly food expenditure was preferred to the use of income because Nigeria still has a huge informal sector whose monthly or annual income might be difficult to measure and food expenditure consumes more than 50% of the income of most families [15].

A key consideration in the creation of SES index using principal components analysis is to include variables that capture inequality between households [16]. There is lack of clear recommendations on the combination of variables that should be included in the SES index. Nonetheless, the use of only the household assets to derive the SES index in our study resulted in clumping and truncation. In truncation, there is an even distribution, but spread over a narrow range, making differentiating between socio-economic groups difficult (e.g. not being able to distinguish between the poor and the very poor) and in clumping or clustering, households are grouped together in a small number of distinct clusters [16]. It was recommended that other methods in solving the problem of truncation and clumping could be to use continuous variables (e.g. the number of acres of land) and using a combination of asset durable ownership, access to utilities and infrastructure, housing characteristics and other variables that appear relevant in assessing household wealth [16]. Hence, adding the per capita food expenditure as a continuous variable that would help to better capture inequality between households (especially given the low variability of the some of the asset variables) helped to solve the problem of truncation.

SES index weights were derived from the eigenvectors of the correlation matrix and the first eigenvector was used because it explains the largest amount of variation in the data [16]. Ownership of fridges had the highest weight (0.46) followed by television (0.43), generator (0.40), car (0.37), electric fan (0.35), air-conditioner (0.27), radio (0.23), per capita weekly food expenditure (0.09), motorcycle (0.02) while bicycle ownership got a negative weighting (−0.15). Combination of these weightings was used to stratify the population into quintiles, Q1 – Q5 with Q1 being the poorest and Q5 being the least poor with approximately 600 people in each. The quintiles were then used to analyse the differences in the payment mechanisms for health services in the population using chi square test of significance. The ratio Q1 to Q5 was used as the measure of equity [17] and shows the gap that has to be covered to ensure equity, a value of 1 signifies perfect equity, a value above 1 signifies that the variable occurs more among the poorest quintile and a value less than 1 signifies that the variable occurs less among the poorest quintile [18]. The ratio is limited by the fact that it fails to measure the experiences of the intermediate quintiles [17].

For the geographic differences new variables were generated and re coded as rural, peri-urban and urban. These were then used to analyse the link between geographic areas of residence of the households with payment and payment coping mechanisms for health services in the population. A rural–urban ratio was calculated and used as a measure of equity similar to the one done for the SES quintiles [9].

Logistic regression was used to examine if there were associations between key dependent variables and some key explanatory variables. The key dependent variables used for the regression were OOPS without reimbursement and use of own money. The explanatory variables used were the SES quintiles, the site groups (rural, peri-urban or urban), age group, sex, main income earner, total number of household residents, whether patient had malaria, whether patient used patent medicine dealer (PMD), whether patient ever attended school and status of patient in household i.e. whether male or female head of household. For some of the continuous variables like age and total number of household residents, new variables were generated and categorised into smaller groups before being put in the regression. The age was categorised into 4 groups which were similar in size <35 = 1, 35-44 = 2, 45-54 = 3 and > =55 = 4. Similarly, since the mean number of household residents was about 5, the variable was categorized into 2 groups of similar size <5 = 1 and > =5 = 2.

## Results

### Socio-demographic characteristics

Most of the respondents were the heads of the households in all the six communities and were mostly male (Table 1). The mean number of household residents was about 5, while the mean age of the respondents ranged from 42 to 49 years. Most of the respondents had some form of education with the mean number of years spent in school being approximately 9 years. Table 1 also shows that radios, televisions and electric fans were the assets owned by most of the respondents. Bicycles and motorcycles were owned by the rural residents more than the peri-urban and urban residents. Ownership of air-conditioners was generally low but like that of cars, there was an increasing trend from rural to urban. Similarly, the per capita weekly food expenditure also shows an increasing trend from the rural to the urban communities.

**Table 1:** Socio-demographic variables and asset ownership

**Variables**	**A1 (Rural) N = 501**	**A2 (Peri-urban) N = 500**	**A3 (Urban) N = 500**	**E1 (Rural) N = 500**	**E2 (Peri-urban) N = 555**	**E3 (Urban) N = 515**
**Status in household**						
**Female household head (%)**	155(31)	137(27)	112(22)	88(18)	64(12)	56(11)
**Male household head (%)**	313(63)	263(53)	334(67)	401(80)	404(73)	180(35)
**Respondent-Main income earner (%)**	462(92)	386(77)	460(92)	480(96)	526(95)	233(45)
**Sex (Male) (%)**	309(62)	270(54)	352(71)	397(81)	403(73)	183(36)
**Ever attended school (%)**	329(66)	434(87)	420(85)	389(82)	509(92)	500(97)
**Mean no of household residents (S.D)**	5(3)	5(5)	5(5)	5(2)	6(4)	6(3)
**Mean age in years of respondent (S.D)**	44(11)	47(15)	45(15)	49(12)	42(12)	42(13)
**Mean no of years spent in school (S.D)**	9(3)	11(4)	10(4)	9(4)	11(4)	13(5)
**Household asset ownership (%)**						
**Radio (%)**	491(98)	490(98)	471(96)	474(95)	548(99)	497(97)
**Television (%)**	405(81)	474(95)	454(91)	351(71)	529(96)	490(95)
**Air-conditioner (%)**	5(1)	20(4)	54(11)	7(1)	38(7)	56(11)
**Bicycle (%)**	141(28)	21(4)	43(9)	146(29)	10(2)	51(10)
**Motorcycle (%)**	131(26)	44(9)	69(14)	141(28)	49(9)	53(10)
**Car (%)**	42(8)	126(25)	145(29)	45(9)	108(20)	211(41)
**Fridge (%)**	212(42)	349(70)	337(68)	188(38)	386(70)	459(89)
**Generator (%)**	128(26)	146(29)	191(38)	121(24)	174(32)	157(31)
**Electric fan (%)**	476(95)	480(96)	473(95)	365(73)	542(98)	506(98)
**Per capita weekly food expenditure in Naira (S.D) US$1 = N150**	399(265)	552(309)	612(986)	376(205)	395(248)	571(259)

A1 = Amansea, A2 = Amawbia, A3 = Awka, E1 = Amokwe, E2 = Iji-Nike, E3 = Uwani.

### Illness patterns

Table 2 shows the number of people in each location that were ill one month prior to interview denoted by N. More than half of those that were ill suffered from malaria. Typhoid fever was also identified to be a main cause of illness suffered by the respondents, followed by diarrhea.

**Table 2:** Illness patterns, where treatment was sought, travel time and treatment cost

**Variables**	**A1 N = 191**	**A2 N = 213**	**A3 N = 162**	**E1 N = 214**	**E2 N = 203**	**E3 N = 159**
**Illness patterns**						
**Had Malaria**	97(51)	106(50)	97(60)	125(58)	131(65)	101(64)
**Had Typhoid**	40(21)	25(12)	30(19)	30(14)	12(6)	18(11)
**Had Diarrhoea**	4(2)	3(1)	7(4)	4(2)	0	1(1)
**Other**	50(26)	79(37)	42(26)	55(26)	64(32)	38(24)
**Where respondents first sought treatment (%)**						
**Traditional Healer*/*Herbalist**	15(8)	10(5)	4(3)	12(6)	9(4)	2(1)
**Patent Medicine Dealer/Chemist**	120(63)	88(41)	82(51)	116(54)	57(28)	81(51)
**Home Treatment**	3(2)	4(2)	14(9)	9(4)	3(2)	5(3)
**Community Health Worker**	1(1)	2(1)	0	2(1)	0	0
**Health Centre**	1(1)	5(3)	0	4(2)	1(1)	0
**Public/General Hospital**	24(13)	38(18)	36(22)	29(14)	29(14)	30(19)
**Private Hospital**	26(14)	58(27)	24(15)	52(24)	47(23)	37(23)
**Others**	1(1)	8(4)	3(2)	2(1)	57(28)	3(2)
**Travel And Treatment Cost (SD)**						
**Mean no of minutes to treatment**	25(40)	27(33)	27(22)	39(34)	21(24)	27(27)
**Mean cost of Transport to treatment**	63(271)	100(295)	107(375)	127(125)	44(101)	82(185)
**Mean drug expenditure**	936(1526)	2840(4935)	2377(10124)	1466(1946)	3237(19451)	2260(4956)
**Mean total Treatment expenditure**	1140(1896)	3527(7711)	2642(10239)	2069(4922)	3758(19600)	3847(9869)

A1 = Amansea (rural), A2 = Amawbia (peri-urban), A3 = Awka (urban), E1 = Amokwe (rural), E2 = Iji-Nike (peri- urban), E3 = Uwani (urban). Note: US$1 = 150 Naira.

Regarding where they first sought treatment, Table 2 shows that PMDs were used in more than 50% of the cases except in the two peri-urban communities. This was followed by private and then public hospitals as the first port of call for treatment. Table 2 also shows a downward trend in the use of both PMD and traditional healers from the rural to the urban dwellers, which shows that the rural dwellers were probably patronizing those low-level providers more than the urban dwellers.

### Travel and treatment cost

Table 2 shows that it took an average of about 26 minutes for each patient to go from home to the place of treatment except in the rural community of Amokwe where it took 39 minutes. The average travel cost for each person in each of the communities was less than US$1 per trip. The drug cost accounted for more than 70% of the total treatment cost for each person, except in the urban community of Uwani where it accounted for about 58%. The total treatment cost for each person ranged from US$7.6 per month in the rural community of Amansea to US$25.6 per month in the urban community of Uwani.

### Payment and payment coping mechanisms used to pay for health services by socio-economic status

Table 3 shows that OOPS without reimbursement was the predominant mechanism used by most people followed distantly by instalment mechanism. The poorest quintile Q1 used OOPS more than any of the other quintiles with an equity ratio of 1.27 though the differences were not statistically significant. The other payment mechanisms like OOPS with reimbursement, health insurance and in-kind were not used so much. Use of own money was the main coping mechanism used by most of the respondents especially the poorest quintile Q1 with an equity ratio of 1.29, though the SES differences were not statistically significant. This was followed distantly by the other mechanisms like use of borrowed money, selling of moveable assets and family land, subsidy, deferred payment, community solidarity and exemption. Generally, none of the payment and payment coping mechanisms showed a statistically significant difference across the five SES quintiles except use of borrowed money with an equity ratio of 14.

**Table 3:** Socio-economic differences in payment and payment coping mechanisms

**Variables**	**Q1 N(%)**	**Q2 N(%)**	**Q3 N(%)**	**Q4 N(%)**	**Q5 N(%)**	**p-value**	**Q1:Q5 ratio**
**Out-of-Pocket but reimbursed**	4(31)	3(22)	1(8)	4(31)	1(8)	0.58	4
**Out-of-Pocket without reimbursement**	236(23)	195(19)	202(20)	200(20)	186(18)	0.50	1.27
**Health Insurance**	1(25)	1(25)	0	0	2(50)	0.41	0.5
**Instalment**	28(28)	17(16)	19(18)	24(23)	16(15)	0.69	1.75
**In-kind**	2(33)	1(17)	1(17)	2(33)	0	0.75	0
**Others**	10(31)	9(29)	7(22)	3(9)	3(9)	0.25	3.33
**Payment coping mechanisms by SES (%)**							
**Own money**	245(23)	202(19)	207(20)	206(20)	190(18)	0.70	1.29
**Borrowed money**	14(46)	5(17)	5(17)	5(17)	1(3)	0.03	14
**Sold moveable assets**	0	0	0	1(100)	0	0.40	0
**Sold family land**	0	0	0	0	0	N/A	0
**Payment subsidized**	12(43)	6(21)	3(11)	2(7)	5(18)	0.09	2.4
**Payment deferred**	3(33)	0	0	3(33)	3(33)	0.20	1
**Community solidarity/someone else paid**	4(21)	4(21)	4(21)	7(37)	0	0.17	0
**Exempted**	3(36)	13)	1(13)	1(13)	2(25)	0.83	1.5
**Others**	1(7)	4(29)	3(21)	1(7)	5(36)	0.20	0.2

### Payment and payment coping mechanisms used to pay for health services by geographic location

Table 4 shows that none of the payment mechanisms was statistically significant by geographic location, though OOPS without reimbursement was used by most of the respondents with a rural urban-ratio of 1.3 indicating some inequity. For the coping mechanisms, use of own money was used by most of the respondents with the urban residents using it less than the peri-urban and rural residents and this was statistically significant with an equity ratio of 1.35. This was followed distantly by other statistically significant coping mechanisms like use of borrowed money, subsidies, deferred payment and community solidarity.

**Table 4:** Geographic differences in payment and coping mechanisms

**Variables**	**Rural**	**Peri-urban**	**Urban**	**p-value**	**R:U ratio**
**Out-of-Pocket but reimbursed**	6(43)	1(7)	7(50)	0.051	0.86
**Out-of-Pocket without reimbursement**	369(36)	382(37)	284(27)	0.24	1.3
**Health Insurance**	2(50)	1(25)	1(25)	0.82	2
**Instalment**	36(34)	45(43)	24(23)	0.28	1.5
**In-kind**	1(17)	1(17)	4(66)	0.11	0.25
**Others**	8(24)	7(21)	18(55)	0.003	0.44
**Payment coping mechanisms by geographic location (%)**					
**Own money**	385(36)	395(37)	286(27)	0.004	1.35
**Borrowed money**	8(26)	7(23)	16(51)	0.01	0.5
**Sold moveable assets**	0	0	1(100)	0.28	0
**Sold family land**	0	0	0	N/A	0
**Payment subsidised**	13(46)	1(4)	14(50)	0.001	0.93
**Payment deferred**	7(78)	1(11)	1(11)	0.03	7
**Community solidarity/someone else paid**	2(11)	12(63)	5(26)	0.03	0.4
**Exempted**	3(37)	1(13)	4(50)	0.26	0.75
**Others**	3(21)	3(21)	8(58)	0.03	0.38

### Regression analysis

The dependent variables were use of OOPS and use of own money because the data shows that these were the variables used by most of the respondents. Unadjusted odds ratio (OR) revealed that use of OOPS had a statistically significant relationship with having malaria (p = 0.036; OR = 1.54) which could be explained by the fact that more than half of those that were ill suffered from malaria. However, adjusted OR as shown in Table 5 revealed that OOPS had a statistically significant relationship with having malaria (p = 0.030; OR = 1.71) and living in an urban area compared to living in a rural area (p = 0.048; OR = 0.53) when controlling for the SES quintile, the total number of household residents, age, sex, main income earner, use of PMD, ever attended school and status in the household.

**Table 5:** Regression analysis of OOPS with explanatory variables

	**Odds ratio**	**Confidence interval**	**p-value**	
**Quintile 1 (base)**	1.00			
**Quintile 2**	0.90	0.46, 1.80	0.778	
**Quintile 3**	1.28	0.59, 2.79	0.533	
**Quintile 4**	1.36	0.62, 2.99	0.438	
**Quintile 5**	1.54	0.67, 3.56	0.314	
**Site group 1 (Rural)**	1.00			
**Site group 2 (Peri-urban)**	0.89	0.49, 1.63	0.703	
**Site group 3 (Urban)**	0.53	0.29, 0.99	0.048	
**Age group 1 (<35)**	1.00			
**Age group 2 (35–44)**	0.60	0.25, 1.42	0.246	
**Age group 3 (45–54)**	1.02	0.41, 2.49	0.973	
**Age group 4 (> = 55)**	0.59	0.25, 1.37	0.218	
**Total number of household residents**	1.61	1.00, 2.61	0.051	
**Sex**	0.74	0.17, 3.22	0.684	
**Main income earner**	1.70	0.69, 4.17	0.248	
**Ever attended school**	1.16	0.61, 2.17	0.654	
**Had malaria**	1.71	1.05, 2.76	0.030	
**Use of Patent Medicine Dealer**	0.21	0.02, 2.06	0.181	
**Status of household head (Male/Female)**	1.41	0.32, 6.19	0.651	
**LR Chi-square**				24.97
**Probability > Chi-square**				0.0704
**Pseudo R^2**				0.0456

Unadjusted OR showed that use of own money had statistically significant relationships with the total number of household residents (p = 0.033; OR = 1.70), whether resident ever attended school (p = 0.021; OR = 1.91), status in the household (p = 0.023; OR = 1.85) and urban residence compared to rural residence (p = 0.009; OR = 0.46). However adjusted OR as shown in Table 6, revealed that use of own money had a statistically significant difference when moving from urban to rural location (p = 0.003; OR = 0.33) after controlling for the SES quintiles, age, sex, main income earner, total number of household residents, having malaria, use of PMD, ever attended school and status in the household. This indicates that urbanites were about 67% less likely to use their own money compared to the rural dwellers after controlling for the potential confounders listed above.

**Table 6:** Regression analysis of use of own money with explanatory variables

	**Odds ratio**	**Confidence interval**	**p-value**	
**Quintile 1 (base)**	1.00			
**Quintile 2**	0.82	0.37, 1.82	0.623	
**Quintile 3**	1.40	0.55, 3.54	0.488	
**Quintile 4**	1.11	0.45, 2.74	0.825	
**Quintile 5**	1.57	0.57, 4.32	0.385	
**Site group 1 (Rural)**	1.00			
**Site group 2 (Peri-urban)**	0.75	0.36, 1.57	0.444	
**Site group 3 (Urban)**	0.33	0.16, 0.69	0.003	
**Age group 1 (<35)**	1.00			
**Age group 2 (35–44)**	0.51	0.16, 1.63	0.253	
**Age group 3 (45–54)**	0.68	0.21, 2.23	0.528	
**Age group 4 (> = 55)**	0.35	0.12, 1.09	0.071	
**Total number of household residents**	1.74	0.98, 3.08	0.058	
**Sex**	1.91	0.36, 10.05	0.447	
**Main income earner**	1.06	0.34, 3.25	0.925	
**Ever attended school**	1.87	0.93, 3.76	0.081	
**Had malaria**	1.08	0.61, 1.91	0.782	
**Use of Patent Medicine Dealer**	0.10	0.01, 1.02	0.052	
**Status of household head (Male/Female)**	0.83	0.15, 4.46	0.828	
**LR Chi-square**				33.79
**Probability > Chi-square**				0.0058
**Pseudo R^2**				0.0792

## Discussion

This study shows that most of the respondents paid for health care using OOPS without reimbursement. It showed a statistically significant relationship between use of OOPS and living in an urban area compared to a rural area. This means that urban dwellers were 47% less likely than rural dwellers to use OOPS to finance health services after controlling for the confounding effects of the variables listed above. This points to inequity in the use of OOPS because in an equitable system, protective mechanisms should be in place to prevent the poor and rural dwellers from such a regressive payment mechanism. This reveals that government efforts to promote pre-payment mechanisms by introduction of NHIS was yet to bear fruits as health insurance was rarely used as seen from this study. Over 90% of the ill respondents reported the use of OOPS in a country where about 64.4% of its citizens live on less than US$1 a day [4], indicating the catastrophic effects of OOPS on the poor people more than the rich. This was also shown in Thailand where OOPS consumed about 2.1% of the annual income of the richest group compared to 21.2% of that of the poorest group [19], while in China, it took 8% and 59% of the income of these groups, respectively [20].

This study showed that use of own money was the coping mechanism used by most of the respondents and was statistically significant when compared between rural and urban dwellers with an equity ratio of 1.35. Further analysis showed that urban dwellers were 67% less likely to use their personal savings to pay for health services compared to rural dwellers who are more likely to be poor which is grossly inequitable. The finding of geographic differences may be because protective mechanisms like insurance as well as borrowing options may be more available to the urbanites than the rural dwellers.

The study did not show any statistically significant difference across the SES quintiles in the use of the payment and payment coping mechanisms though the equity ratios were high indicating some inequity. This finding may be because the prevailing low utilisation of prepayment mechanisms like insurance and the absence of mechanisms other than OOPS, leave most of the Nigerian population with no other option than to use their personal savings for health care payments.

The finding that drug cost took up about 70% of the mean treatment expenditure for most of the households means that even if user fees are waived for services like registration and consultation, most of the poor people would still have to bear a huge chunk of the direct cost by buying drugs themselves which could be catastrophic to the poor. This is similar to what was seen in Ghana and India where drugs cost took up over 60% of the cost of treating malaria [21] and lymphatic filariasis respectively [22]. Hence, any government policy that will offer free drugs, drug subsidies, vouchers and duty waivers (for those that want to import drugs) will tackle the financial burden of health cost. The policy may encourage drug production by local manufacturers by offering loans and tax cuts thereby driving down the cost of these drugs.

The finding that rural dwellers in the two states spent less out of the total treatment expenditure, could be because they did not have the money to pay for health services or that they patronised low-level providers who charged less in most cases [18]. Studies in Tajikistan showed that health care utilization differed across SES groups according to ability to pay and showed that OOPS prevented poor people from seeking care and prevented those that did from receiving appropriate care [23,24]. These studies also showed that these barriers to access could be reduced by health care funding from public sources, paying family doctors incentives to reduce the payment by the patients and provision of free drugs at health facilities.

This study showed that malaria was the main illness suffered by most of the respondents buttressing the fact that it is one of the main causes of morbidity and mortality in Nigeria accounting for 50% of outpatient consultations, 30% of childhood mortality and 11% of maternal mortality [25]. It also showed that traditional healers were used more than health centres and community health workers even though health centres are meant to be the primary place of treatment for most public health illnesses, being the lowest in the three tiers of health service provision by the government, and traditional healers have not yet been shown to be properly regulated to give top quality treatment.

One limitation of the study was the methodology and variables that were used to construct the SES index using principal components analysis. This is because of the low variability of the asset variables and the resultant clumping and clustering, which were solved by the addition of per capita food expenditure to the index. However, the addition of the food expenditure variable may not be ideal, but there were no other SES discriminatory variables that we had data on. Therefore, although we ended up with smooth distribution of SES quintiles, future studies should ensure that they collect data on many continuous variables other than expenditure variables that can be used to solve the problems of clumping and truncation of the SES index.

## Conclusion

Overall, this study shows gross geographic inequities in the payment and payment coping mechanisms for health services. Though the SES differences were not statistically significant, they showed high equity ratios indicating more use among poor and rural dwellers. The high expenditure incurred on drugs alone highlights the need for expediting pro-poor interventions like exemptions and waivers aimed at improving access to health care for the vulnerable poor and rural dwellers. The observed inequities could be addressed by strict implementation of pro-poor measures like exemptions and waivers to bridge the rich-poor and urban–rural gap thereby increasing healthcare utilisation and decreasing cost.

## Competing interests

The authors declare that they have no competing interests.

## Authors’ contributions

OO conceived the study. CO coordinated the study. OE wrote the first draft of the manuscript. All the authors contributed to the research study design, data acquisition, data collection, data analysis, data interpretation, manuscript drafting and revision for important intellectual content. All authors read and approved the final manuscript.

## Pre-publication history

The pre-publication history for this paper can be accessed here:

http://www.biomedcentral.com/1472-6963/13/87/prepub
